# Supplementary material for: Causal Relationship between Aging and Anorexia Nervosa: A White-Matter-Microstructure-Mediated Mendelian Randomization Analysis
Source: Biomedicines. 2024 Aug 16;12(8):1874. doi: 10.3390/biomedicines12081874 (PMC11351342; doi:10.3390/biomedicines12081874)
Supplement: Supplementary file 1 [file biomedicines-12-01874-s001.zip › Supplementary Figure/Figure S4/Singe SNP plot of MR analysis for TL to ubm-b-1530.pdf]

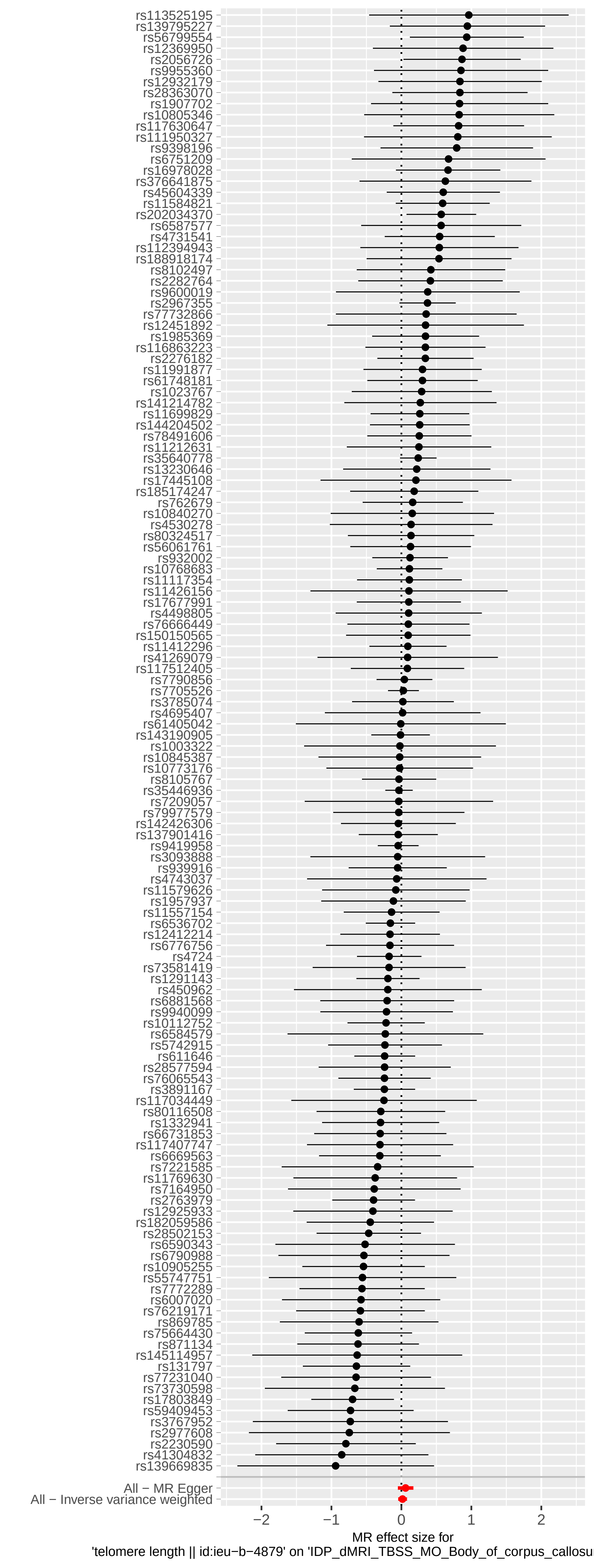
rs113525195
rs139795227
rs56799554
rs12369950
rs2056726
rs9955360
rs12932179
rs28363070
rs1907702
rs10805346
rs117630647
rs111950327
rs9398196
rs6751209
rs16978028
rs376641875
rs45604339
rs11584821
rs202034370
rs6587577
rs4731541
rs112394943
rs188918174
rs8102497
rs2282764
rs9600019
rs2967355
rs77732866
rs12451892
rs1985369
rs116863223
rs2276182
rs11991877
rs61748181
rs1023767
rs141214782
rs11699829
rs144204502
rs78491606
rs11212631
rs35640778
rs13230646
rs17445108
rs185174247
rs762679
rs10840270
rs4530278
rs80324517
rs56061761
rs932002
rs10768683
rs11117354
rs11426156
rs17677991
rs4498805
rs76666449
rs150150565
rs11412296
rs41269079
rs117512405
rs7790856
rs7705526
rs3785074
rs4695407
rs61405042
rs143190905
rs1003322
rs10845387
rs10773176
rs8105767
rs35446936
rs7209057
rs79977579
rs142426306
rs137901416
rs9419958
rs3093888
rs939916
rs4743037
rs11579626
rs1957937
rs11557154
rs6536702
rs12412214
rs6776756
rs4724
rs73581419
rs1291143
rs450962
rs6881568
rs9940099
rs10112752
rs6584579
rs5742915
rs611646
rs28577594
rs76065543
rs3891167
rs117034449
rs80116508
rs1332941
rs66731853
rs117407747
rs6669563
rs7221585
rs11769630
rs7164950
rs2763979
rs12925933
rs182059586
rs28502153
rs6590343
rs6790988
rs10905255
rs55747751
rs7772289
rs6007020
rs76219171
rs869785
rs75664430
rs871134
rs145114957
rs131797
rs77231040
rs73730598
rs17803849
rs59409453
rs3767952
rs2977608
rs2230590
rs41304832
rs139669835
All − MR Egger
All − Inverse variance weighted
−2
−1
0
1
2
MR effect size for
'telomere length || id:ieu−b−4879' on 'IDP_dMRI_TBSS_MO_Body_of_corpus_callosu
